# *In-Situ* Monitoring of Real-Time Loop-Mediated Isothermal Amplification with QCM: Detecting *Listeria monocytogenes*

**DOI:** 10.3390/bios11090308

**Published:** 2021-08-31

**Authors:** Sirirat Wachiralurpan, Isaratat Phung-On, Narong Chanlek, Supatra Areekit, Kosum Chansiri, Peter A. Lieberzeit

**Affiliations:** 1 Maintenance Technology Center, Institute for Scientific and Technological Research and Services, King Mongkut’s University of Technology Thonburi, 126 Prachautit Rd., Bangkok 10140, Thailand; isaratat.phu@kmutt.ac.th; 2 Synchrotron Light Research Institute (Public Organization), 111 University Avenue, Muang District, Nakhon Ratchasima 30000, Thailand; narong@slri.or.th; 3 Innovative Learning Center, Srinakharinwirot University, Sukhumvit 23, Bangkok 10110, Thailand; supatraa@g.swu.ac.th; 4 Center of Excellence in Biosensors, Panyananthaphikkhu Chonprathan Medical Center, Srinakharinwirot University, 222 Moo1 Tiwanon Rd., Pakkred District, Nonthaburi 11120, Thailand; prof.kosum@gmail.com; 5 Department of Biochemistry, Faculty of Medicine, Srinakharinwirot University, Sukhumvit 23, Bangkok 10110, Thailand; 6 Department of Physical Chemistry, Faculty for Chemistry, University of Vienna, Waehringer Strasse 42, 1090 Vienna, Austria

**Keywords:** *Listeria monocytogenes* (*L. monocytogenes*), *hly* gene, real-time loop-mediated isothermal amplification (real-time LAMP), quartz crystal microbalance (QCM), X-ray photoelectron spectroscopy (XPS), LAMP products analysis

## Abstract

Functionalized DNA sequences are promising sensing elements to combine with transducers for bio-sensing specific target microbes. As an application example, this paper demonstrates in situ detection of loop-mediated isothermal amplification products by hybridizing them with thiolated-ssDNA covalently anchored on the electrodes of a quartz crystal microbalance (QCM). Such hybridization leads to a frequency signal, which is suitable for monitoring real-time LAMP amplification based on mass-sensing: it detects interactions between the complementary nucleobases of LAMP products in solution and the thiolated-ssDNA probe sequence on the gold surface. Target DNA LAMP products cause irreversible frequency shifts on the QCM surfaces during hybridization in the kHz range, which result from both changes in mass and charge on the electrode surface. In order to confirm the LAMP assay working in the QCM sensing system at elevated temperature, the sky blue of positive LAMP products solution was achieved by using the Hydroxy Naphthol Blue (HNB) and agarose gel electrophoresis. Since on-QCM sensing of DNA hybridization leads to irreversible sensor responses, this work shows characterization by X-ray photoelectron spectroscopy (XPS) core spectra of S2p, N1s, Mg1s, P2p and C1s. XPS results confirmed that indeed both DNA and by-products of LAMP attached to the surface. *Listeria monocytogenes* DNA served to study *in-situ* detection of amplified LAMP products on DNA-functionalized surfaces.

## 1. Introduction

Detection of DNA-based immobilization and hybridization on solid surfaces is the basis of several biotechnological tools and strategies, including DNA microarrays [1,2] and biosensors [3,4]. Previous research on DNA-modified surfaces has largely explored functionalized gold [5] and other metals [6]. Immobilizing single-strand capture DNA on electronic devices and circuits requires chemical modification that leads to the desired recognition structures (i.e., self-assembled monolayers) [7,8]. A recent study has shown that using a spacer between immobilized DNA strands, such as L-cysteine, increases sensitivity, because it makes the capture probes on the surface sterically more accessible [9] to the analyte. Herein, we demonstrate how the results of that paper can be carried forward to ensure amplification and measurements in one step simultaneously, i.e., *in-situ*. This both reduces the time for the experiments and allows for semiquantitative assessment of DNA contamination. 

Among DNA amplification methods, loop mediated isothermal amplification (LAMP) has a number of potential advantages for amplifying signals of low numbers of target DNA [10], such as operating at constant temperature and being easy to perform in a simple thermostatically controlled heater block [11]. This makes it highly cost-efficient. Furthermore, it uses a series of two (or three) primer pairs binding to six (or eight) positions of the target sequence with high specificity [12,13], eliminating time loss and yielding comparably large polynucleotides [13,14]. Bringing such target DNA close to electrode surfaces increases both mass and the local charge density via the negative charge of the phosphate backbone and thus makes it potentially accessible for sensing purposes. Relatively few papers discuss high-resolution surface analytical techniques [15,16,17,18,19,20] to characterize DNA self-assembly on surfaces. 

**Scheme 1:**
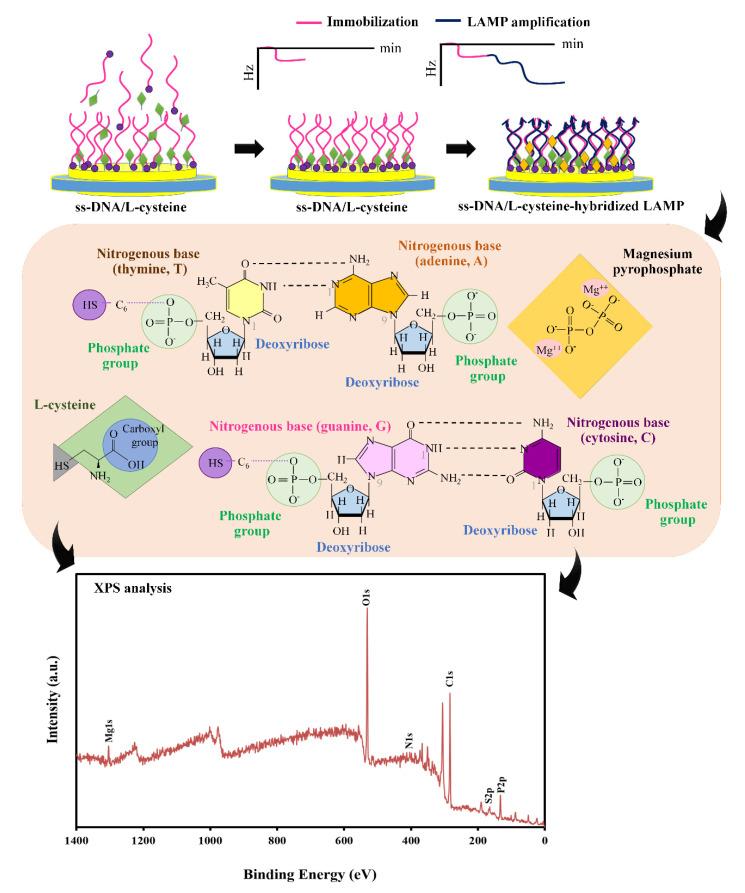
Scheme of real-time LAMP-QCM sensor approach.

For that purpose, we use a recently developed method to provide *in-situ* measurement of DNA amplification [9], relying on QCM measurements (Scheme 1). That paper demonstrates the method for achieving such measurements in principle and strongly focuses on the optimization steps required to ensure optimal hybridization between the immobilized oligonucleotide and a target single-strand DNA. It therefore laid out the fundaments of how to prepare optimal self-assembled monolayers for that purpose. Herein, we investigate how to actually apply such a system directly in an amplification cocktail. There, the QCM operates at isothermal conditions, namely at the temperature for amplification and probe-target hybridization, rather than injecting samples into a detection chamber offline. This allows us to simultaneously monitor LAMP activity and diagnose *Listeria monocytogenes* on the sensor. To develop a more in-depth understanding of DNA hybridization and the efficiency of LAMP target capture, we herein characterized the surfaces after binding with XPS. This approach allows for validating the mass signals on QCM and for quantifying the chemical composition of the surface layer at different stages of recognition/sensing. Combining the results of the two approaches leads to a more comprehensive picture of the self-assembled monolayers on the device surfaces during the amplification-hybridization assay.

## 2. Materials and Methods

### 2.1. Chemicals

We used a patented 5′-end thiol-modified ssDNA probe (patent submission number 1601004782) as the recognition species on QCM electrodes. It specifically binds to the nucleotide sequence of the *hly* gene of *L. monocytogenes*. The respective single-stranded DNA comprises 32 oligomers, including 6 nucleosides of adenine, guanine and cytosine each, and 14 thymine nucleosides in order to design a single gene target DNA probe; this probe was synthesized by Bio Basic Inc., Canada. To form the final recognition layer, we co-immobilized DNA capture probes with L-Cysteine (Merck, Darmstadt, Germany) to increase the probe–probe distance on QCM electrodes. In brief, we prepared the immobilization solution by mixing 10 µL of 100 μM ssDNA-SH probe with 30 µL of 0.1% (w/v) L-cysteine and added running buffer (0.1 mM phosphate-buffered saline (PBS), pH = 7.4) to a volume of 200 µL. To prepare 0.01 M phosphate-buffered saline (0.01 M, PBS), we dissolved 8.5 g sodium chloride (NaCl; purchased from Applichem, Darmstadt, Germany), 0.2 g sodium dihydrogen phosphate and 1.4 g di-sodium hydrogen phosphate (NaH_2_PO_4_ and Na_2_HPO_4_; both purchased from Merck, Darmstadt, Germany in the highest available purity) in almost 1 L of deionized water and then adjusted to desired pH = 7.4 followed by filling up with water to 1 L. The running buffer (0.1 mM PBS, pH 7.4) used in this study was diluted from 0.01 M PBS by deionized water, then adjusted to the desired pH of 7.4. Brilliant gold paste (gold colloid, 10% gold content) purchased from Heraeus, Germany, was used for screen-printing the QCM electrode patterns on a quartz plate. Hydroxy naphthol blue (HNB) purchased from Sigma-Aldrich (Burlington, MA, USA), was used for easy investigation of LAMP products. Sterile deionized water (18.2 MΩ cm^−1^) served for preparing all solutions.

### 2.2. Template DNA Isolation

The Department of Medical Science, Ministry of Public Health, Thailand (DMST) provided the *L. monocytogenes* DMST 17303 sample for extracting the template DNA. In detail, one colony of pure culture was picked and inoculated into 10 mL brain heart infusion (BHI) broth (BBL, Becton Dickinson Microbiology Systems, Cockeysville, MD, USA) followed by growing overnight at 37 °C with 250 rpm shaking speed. Bacterial cell harvesting started with centrifugation of the aforementioned culture broth by using a fixed-angle rotor (Hettich GmbH and Co. KG, Tuttlingen, Baden-Württemberg, Germany) at 13,000 rpm for 10 min and discarding of the supernatant. The pellet cells were washed thrice by adding 1 mL of sterile deionized water. During washing, the bacterial cells were suspended gently, followed by harvesting. Then, pellet cells were re-suspended in 0.1 mL sterile deionized water. Heating the cells to 100 °C in a heating box served to physically break them. Incubation time for heating and then releasing the genomic DNA from *L. monocytogenes* colonies was set to ten minutes. The suspension was processed by centrifuging at 13,000 rpm for 1 min, then carefully carrying over the supernatant to a new microcentrifuge tube. The concentration of *L. monocytogenes* DMST 17303 DNA was determined by using a NanoDrop^TM^ 2000 UV-vis Spectrophotometer (Thermo Scientific, Waltham, MA, USA). Template DNA was diluted with sterile deionized water to a final concentration of 400 ng/µL and used as working DNA for the LAMP procedure on the real-time QCM/DNA approach.

### 2.3. QCM Sensor Measurements and Construction of ssDNA Films

Details of the QCM sensor set-up and fabrication of the sensing electrode can be found in a previously published paper [9], which demonstrates all the necessary optimization experiments to achieve ideal self-assembled monolayers containing an oligonucleotide capture probe. In brief, we printed the electrode structures on both sides of AT-cut single crystal quartz plates with 13.8 mm diameter and 168 µm thickness with brilliant gold paste to yield QCM. To reveal the gold electrodes, QCM were burned in the oven at 400 °C for 2 h after printing.

Modified QCM were placed in a custom-made PDMS cell for measurements. The temperature was kept at 60 °C by a thermostat (Thermo Haake^®^ DC30, Gebrüder HAAKE GmbH, Karlsruhe, Germany), which turned out the optimal temperature for LAMP amplification [21] when developing a colorimetric test for a different gene of *L. monocytogenes*. This cell was then connected to a custom-made oscillator circuit to operate the device. A frequency counter monitored the corresponding resonant frequency and transferred it to a computer via a custom-made LabView routine.

Measurements were carried out in stopped flow mode: first, we flushed the cell with running buffer (0.1 mM PBS, pH 7.4) and then waited for the frequency reaching equilibrium signal, i.e., the base line. Then, we immobilized a mixed layer of ssDNA probes and L-cysteine molecules on the gold electrodes by carefully introducing the respective mixed solution (5 μM ssDNA probe, 0.015% L-cysteine, 0.1 mM PBS (pH 7.4)). We followed immobilization directly by recording the changes in frequency *in-situ*. The ssDNA probe and L-cysteine molecules were randomly immobilized on the electrode surface for 60 min, followed by washing off unbound molecules. During washing steps, we stopped the frequency readout and re-started it after having injected the next sample into the measuring cell.

### 2.4. Real-Time LAMP Monitoring with QCM

After immobilizing the self-assembled ssDNA film on the device surface, we injected the LAMP mixture containing approximately 6.4 μg *L. monocytogenes* DNA (positive reaction) to generate the desired LAMP products on QCM electrodes within 60 min. The LAMP mixture in total 200 μL consisted of 2 μM LAMP inner primer, 0.2 μM LAMP outer primer, 1.6 mM dNTPs (New England Biolabs, Ipswich, MA, USA), 0.5 M Betaine (Sigma-Aldrich, USA), 5.6 mM MgSO_4_ (New England Biolabs, USA), 8U *Bst* DNA polymerase (New England Biolabs, USA) and 1X of ThermoPol^®^ reaction buffer. After roughly 60 min, the measurement was paused to flush the quartz three times. In order to monitor LAMP in real-time, the resonance frequency of LAMP amplification and hybridization signals were recorded as a function of time.

### 2.5. Visual Inspection of LAMP Products

The LAMP mixture used here is the result of previously published optimization [21], which revealed that one can use the turbidity signal at 650 nm as a measure of determining how far the LAMP process has proceeded. It makes use of the increasing amount of white magnesium pyrophosphate precipitate in the solution. Negative control (without template DNA) was analyzed to verify that the LAMP mixture does not contain DNA leading to positive results. Two percent agarose gel electrophoresis with ethidium bromide staining following a previously published protocol [22] served to confirm ladder-like pattern of LAMP products. 

In parallel, a colorimetry approach served to screen the LAMP products, namely Hydroxy Naphthol Blue (HNB) staining [23,24,25,26]. HNB was dissolved in sterile deionized water to yield 1 mM solutions that exhibit a cherry-red color. This working reagent served to determine color differences between negative test solutions (without template DNA) and positive sample/standard solutions visually. For that purpose, we added 3 μL of 1 mM HNB solution to each LAMP reaction batch before starting the experiment. This ensured the optimal concentration of 0.12 mM HNB in the LAMP reaction solution. After successful LAMP amplification of the target DNA, the colors of solutions changed.

In order to determine the different colors in a more quantifiable manner, 200 µL LAMP-HNBs (negative and positive ones) were each pipetted into a sterile 96-well plate (Thermo Scientific, Rochester, NY, USA). A microplate spectrophotometer (Epoch, BioTek^®^, Winooski, VT, USA) with Gen5 version 2.01 microplate data analysis software (BioTek^®^) served for recording the respective absorbance spectra.

### 2.6. Assessing QCM Electrode Sensitivity toward LAMP Product

For assessing the sensitivity of the QCM sensors toward the target DNA, LAMP amplification using different concentrations of *L. monocytogenes* DMST 17303 gDNA were carried out. Samples contained approximately 0.8 ng to 6400 ng (6.4 μg) target DNA in 200 µL solution at the beginning time of LAMP amplification. Again, the QCM read out the frequency shifts resulting from these different concentrations to calibrate the system.

### 2.7. QCM Surface Characterization 

XPS measurements of LAMP amplification products hybridized on QCM electrodes took place on a PHI5000 VersaProbe II Scanning XPS Microprobe (ULVACPHI, Japan) equipped with a high resolution 180° hemispherical electron energy analyzer and a quartz crystal monochromatic AlK_α_ (1486.6 eV) X-ray as the excitation source with 100 µm^2^ beam area. This instrument is located at Synchrotron Light Research Institute (SLRI) beamline 5.3 (SUT-NANOTEC-SLRI XPS) building, Thailand, but does not use the beamline itself. All XPS measurements were carried out at room temperature in ultra-high vacuum (UHV) with a base pressure of 10^−7^ mbar. High-resolution scans were acquired using a 45° takeoff angle for the peak regions typical for S2p, N1s, Mg1s, P2p and C1s. For energy calibration, all binding energies were calibrated relative to the Au 4f_7/2_ peak of a reference gold sample at a binding energy of 83.90 eV. Several points at the surface of each sample were studied for checking the uniformity of the samples. No significant changes in XPS spectra were observed in repeated scans, indicating that the DNA samples were rather stable under X-ray flux. The spectra were fitted using Gaussian−Lorentzian functions by the Multipak software with simultaneous background optimization. All binding energies of the PE (photoemission) peaks are given with an accuracy of ±0.5 eV. Quantification, reported as the relative elemental percentage, was carried out by using the integrated area of the fitted core lines, after Shirley background subtraction, and by correcting for the atomic sensitivity factors.

## 3. Results and Discussion

### 3.1. Sensor Functionalization and ssDNA/L-Cysteine Coverage Measurement

**Figure 1:**
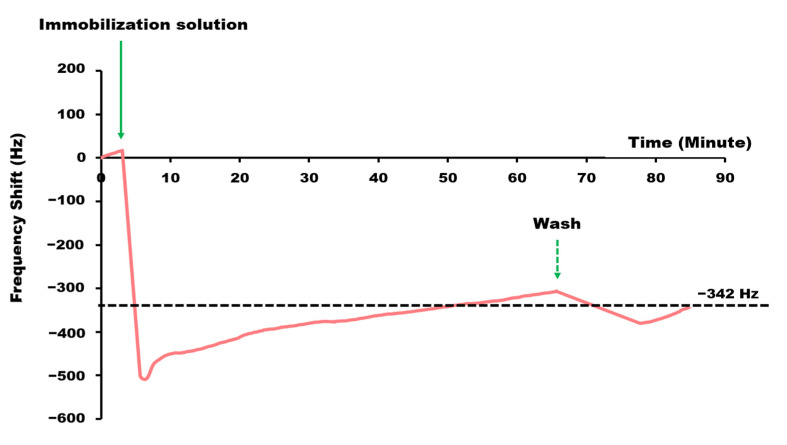
QCM resonant frequency shift upon immobilization of thiolated-ssDNA probe with L-cysteine on the QCM electrode.

Figure 1 shows the QCM frequency shift when exposing it to the mixture containing thiolated-ssDNA and L-cysteine. Clearly, the frequency drops after exposure and reaches the equilibrium value after roughly one hour. The reason for the exact shape of the response curve is not fully clear but most probably a consequence of the fact that DNA molecules come with very high charge density, which of course influences the signals [27,28,29,30]. Furthermore, the response behavior also demonstrates irreversible, covalent binding of the species to the electrode surface: the signal remains constant after washing. 

The irreversible signal (*ΔF*, Hz) can be converted into a mass change on the quartz crystal and electrode by applying Sauerbrey’s equation [31], which is given in Equation (1)

(1) $$\Delta F=-\frac{2F0^{2}}{A\sqrt{\rho_{q}\mu_{q}}}\;\Delta m$$

where Δ*F* is the measured irreversible frequency shift, Δ*m* the mass change (g), *A* the electrode area 0.2 cm^2^, $\rho_{q}$ the density of quartz (2.648 g·cm^−3^) and $\mu_{q}$ the shear modulus of quartz for AT-cut crystal (2.947 × 10^11^ g·cm^−1^·s^−2^) so that $S_{Q}$ or $\rho_{q}\mu_{q}$ is 2.26 × 10^8^ cm^2^ g^−1^ s^−1^ and 10 MHz AT-cut Quartz was used in the study.

Inserting the respective values into the Sauerbrey’s equation at the irreversible frequency shift (−342 Hz with noise being ~5 Hz) led to estimate the mass change after immobilization on the QCM electrode as approximately 300 ng. According to previous work [9], the molecular ratio in the solution is roughly 1 molecule ssDNA probe per 55 molecules L-cysteine. The hybridization sensor functionalized with thiolated single-stranded DNA probes thus contains approximately 1.8 × 10^13^ DNA molecules (MW = 9982.52 g/mol) (see Supplementary file S1) when co-immobilizing them with L-cysteine.

### 3.2. Real-Time LAMP-QCM Measurement

**Figure 2:**
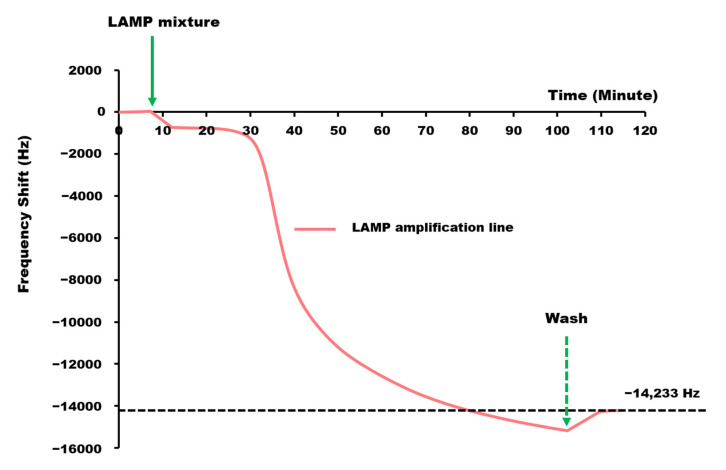
Real-time QCM resonant frequency shift with irreversible signal of LAMP products adsorbed on QCM electrode.

Figure 2 shows the QCM sensor responses obtained while carrying out the LAMP amplification reaction directly in the measuring cell. As LAMP is an isothermal process, it turned out that placing the measuring cell inside a Styrofoam box for temperature insulation is sufficient for that purpose. 

Immediately after injecting the LAMP mixture into the measuring cell, the frequency decreases by almost 1 kHz. The reasons for this are most probably that the electrostatic environment around the electrode surface changes, and non-specific adsorption on the surface occurs. After some 20 min, the signal starts decreasing further and reaches a value of roughly −15 kHz. After washing, the signal remains about 14.2 kHz lower than the starting value, which clearly demonstrates substantial binding: this irreversible signal results from hybridization of LAMP amplification products with the probes on the device surface. The frequency shift observed corresponds to a mass change of approximately 12.6 μg LAMP amplification products leading to around 7.7 × 10^14^ copies of the target gene on the surface and corresponded to total nucleotides molecules of 7.3 × 10^14^ molecules. However, the number of irreversibly bound LAMP product nucleotides cannot exceed the number of capture probes (1.8 × 10^13^, see above). Therefore, the data suggest that amplified polynucleotides on average contain around 40 copies of the target DNA sequence.

### 3.3. Rapid Screening of LAMP Products

**Figure 3:**
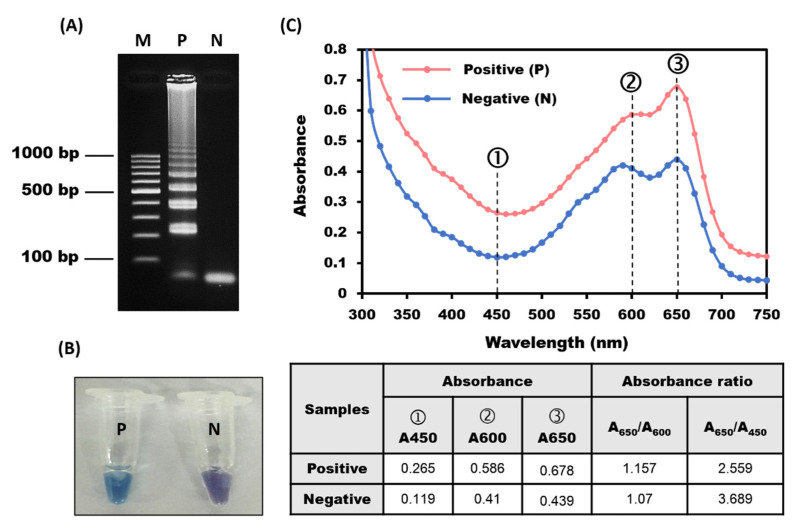
(**A**) LAMP profiles on 2% agarose gel electrophoresis. (**B**) Direct visualization under natural light and (**C**) spectra of LAMP with 0.12 mM hydroxy naphthol blue reaction. M, GeneRuler™ 100 bp DNA ladder marker (Thermo Scientific, USA); P, positive control; N, negative control (without DNA template).

In routine, to confirm the LAMP reaction was amplified at the total volume of 25 µL, 2 µL of LAMP positive control and LAMP negative control, respectively, were loaded well by well in 2% agarose gel. After staining, the LAMP pattern shows DNA ladder products of upper 200 bp compared to the GeneRuler™ 100 bp DNA ladder marker (Thermo Scientific, USA) (Figure 3A). To validate this result, samples containing the same LAMP mixture with HNB added to a total volume of 200 µL of LAMP-HNB mixture (eight times the volume of the 25 µL LAMP mixture) were incubated at the same conditions as the measuring cell. Evidently, the color of the solution changes: the positive control appears intense blue, while the negative control turns out purple. The corresponding UV-Vis spectra reveal that the color change corresponds to changes in spectral features: colors of both positive and negative samples were characterized by the absorbance ratios A_650_/A_600_ and A_650_/A_450_ (with the subscripts denoting the respective wavelength in nm), respectively. This covers both the shapes and heights of the two spectral bands and thus is a reasonable measure for the color change. The LAMP positive control reveals a higher A_650_/A_600_ ratio, than the negative control, namely 1.2 (1.157) as compared to 1.1 (1.070), respectively. The A_650_/A_450_ ratios are approximately 2.6 for positive control and 3.7 for negative control, respectively (see Figure 3B,C).

### 3.4. Estimating Bacteria Load of the Sample

**Figure 4:**
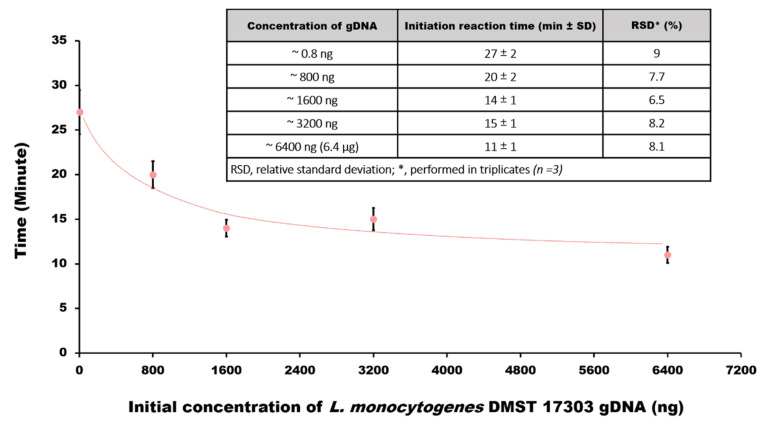
Time for onset of QCM frequency response as a function of initial concentration of target DNA.

In the same way as other amplification strategies, LAMP of course does not allow for (precise) quantitative analysis. However, measuring amplification *in-situ* actually opens up a way for at least reasonable estimating the bacteria load of a contaminated sample: as long as the concentration of the target analyte is lower than the lower limit of detection (LoD) of the QCM sensor, there should be no signal visible. This only changes once the concentration of the target product has reached that value. Therefore, the time between injecting a sample and the onset of the frequency decrease due to binding of the LAMP product should be indicative of the initial sample concentration. To assess this hypothesis, we tested five different initial concentrations of *L. monocytogenes* DMST 17303 gDNA samples. Figure 4 shows the results: when injecting a LAMP mixture containing approximately 0.8 ng of *L. monocytogenes* gDNA, starting amplification led to a signal onset after 27 min It is worth noting that this clearly showcases an added advantage of the QCM-based assay: turbidity measurements at 650 nm showed a signal only after 46 min [21]. Switching to measuring on the QCM in situ hence improves sensitivity by decreasing the measuring time. Increasing the initial amount in the mixture to 800 ng gDNA reduces the signal onset time to 20 min. At the highest amount tested—6400 ng—one can distinguish the signal after 11 min. According to literature, precision at below 20% [32] confidence interval is acceptable for such measurements. The onset times of this QCM system show relative standard deviations from 6% to 10% of the working range of initial DNA concentration and thus meet this requirement. The trend line also shows a clear correlation between the onset times and initial concentrations of *L. monocytogenes* gDNA. *In-situ* QCM measurements, especially in the lower concentration range, i.e., until adding up to 800 ng initial DNA to the sample, reveal that the onset time of the frequency shift very strongly depends on the initial concentration. Above that, the signal basically saturates. Given that legislation for ready-to-eat food strictly requires a “zero tolerance” policy of *Listeria monocytogenes* contamination, the sensor meets the legal requirements. However, it goes even beyond them by assessing if bacteria contamination is low or high. Despite not being of regulatory value, it still adds a piece of information that may be useful for elucidating the possible source of contamination. 

### 3.5. X-ray Photoelectron Spectroscopy (XPS) Spectra

Thus, real-time QCM measurements clearly demonstrate that LAMP amplification products hybridize with the capture probes on the surface, causing substantial irreversible frequency signals. To test this with an independent technique, we applied XPS measurements to confirm compositions of the respective self-assembled layers on the surfaces. As previously stated, those comprise a mixture of ssDNA capture probe and L-cysteine as a spacer immobilized on the electrodes plus any amplification product bound to it. Previous reports for related systems include measurements on adsorbed DNA bases [33] and, more recently, limited results for ssDNA films [16,34]. 

**Figure 5:**
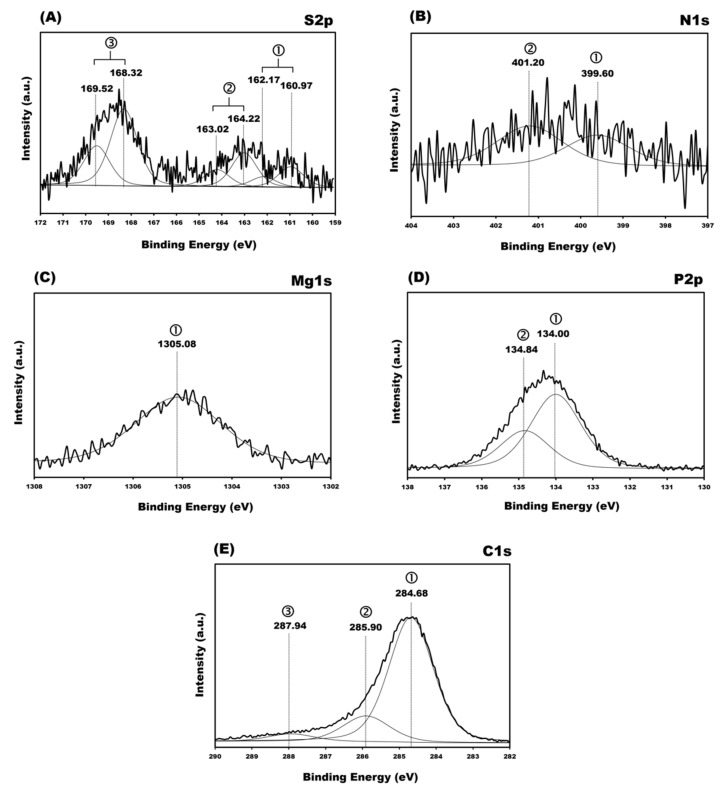
High-resolution XPS spectra of L-cysteine/ssDNA hybridize to the target sequence of LAMP amplification products on Au electrode. (**A**) S2p, (**B**) N1s, (**C**) Mg1s, (**D**) P2p and (**E**) C1s.

Figure 5 and Table 1 show the high-resolution XPS data for all five principal element signals, namely, S2p, N1s, Mg1s, P2p and C1s of the products on the adsorbed surface. Given the mixture of thiolated nucleotides and L-cysteine, one of course expects signals from sulfur originating from each species directly bound to gold. The fitted curves of deconvoluted S2p signals reveal (comparably weak) peaks at 160.97, 162.17, 163.02, 164.22, 168.32, and 169.52 eV, respectively, as shown in Figure 5A. Two S2p signals comprise of 2p_3/2_ and 2p_1/2_ with an area intensity ratio of 2:1 and spin-orbital splitting of ΔBE = 1.2 eV: the sub-spectrum at a binding energy (BE) of 160.97 eV is attributed to sulfur bound to the gold surface (Au-S). A second and third doublet shifted to higher BE at 163.02 and 168.32 eV, respectively, can be assigned to thiol (S-H) and oxidized sulfur species on the surface [19,34,35,36,37,38]. The minor components at low BE most likely correspond to transitions between physisorbed to chemisorbed *sulfur* species. Due to the complexity of the products on the surface, it is not surprising to find both the physisorbed state (thiol molecules) and transition to chemisorbed thiolates [39,40,41]. The physisorbed state comprises molecules “lying” flat on the surface [40,42] stabilized by van der Waals interactions between the molecular species and the surface.

LAMP amplification generates stem loop DNA with various stem lengths and structures containing multiple loops. It also releases the reaction by-product, magnesium pyrophosphate. Thus, one can confirm hybridization of target DNA LAMP products with ssDNA probes on the QCM surface by the presence of large amounts of nitrogen from nucleobases and phosphorus from phosphate originating from both the DNA backbone and magnesium pyrophosphate (by-product of LAMP) [14]. Figure 5B shows that the N1s spectrum resolves into two peak components with BEs of 399.60 and 401.20 eV. The higher BE peak at 401.20 eV corresponds to N1s from cytosine and adenine [43], to N–(C=O)–N from thymine of double-stranded DNA, and to -NH_2_ from L-cysteine. The peak at the lower BE peak is attributed to conjugated –N= [43]. 

Phosphorous from the phosphate backbone of double-stranded DNA and magnesium pyrophosphate also indicate successful hybridization. Deconvoluting the fit in the P2p signal range reveals a doublet with BEs of 134.84 and 134.00 eV (Figure 5D). It consists of 2p_1/2_ and 2p_3/2_ because of spin-orbital splitting of ΔBE = 0.84 eV [19] with an assumed area intensity ratio of 1:2. Cho et al., 2012 [44] reported that a peak at ~134 eV corresponds to phosphorus in the DNA backbone immobilized on an III–V semiconductor. 

Figure 5C shows Mg1s spectra at BE of 1305.08 eV, which was ascribed from magnesium pyrophosphate [45] present on the non-selective area of the QCM surface after the LAMP amplification.

Finally, the signals in the C1s region after fitting reveal three underlying components (Figure 5E). The strongest signal centered at 284.68 eV, includes –C–C– (glycosidic carbon), –C= (aromatic carbon C5 of cytosine and thymine), –CH (alkyl chain), and –CH_3_ of methyl groups. It might also include carbons of aliphatic contaminations. The peak at 285.90 eV can be assigned to C–N, N– C=N (carbon bound to nitrogen) and C–O (carbon of sugar groups and sugar–phosphate bonds). The peak at the higher BE of 287.94 eV corresponds to carbonyl groups in thymine and cytosine, i.e., N–(CO)–N, N–(CO)–C.

The single-stranded oligonucleotides containing 32 bases either are not stretched or are oriented away from the surface normal, because they capture the target LAMP amplification product, leading to an estimated total number of nucleotides on the QCM surface being 7.3 × 10^14^ molecules (calculated from the DNA formula without 5′ monophosphate). The layer of real-time LAMP amplification seems to be more densely packed than the layers of the diluted hybridized LAMP products, which is in line with previous observations [9,38].

## 4. Conclusions

LAMP-QCM brings together state-of-the-art research from both biochemistry and analytical measuring science. This work demonstrates a systematic study for LAMP-QCM from preparation, chemical reaction, electronic response and characterization by the use of two typical co-immobilization (a thiolated-ssDNA probe and L-cysteine) and the LAMP mixture solution as proof-of-concept confirmed by XPS analysis of the surface. The results suggest that LAMP-QCM constitutes an interesting alternative to existing bioassays for real-time monitoring of target DNA, in this case of *Listeria monocytogenes,* for instance, to ensure food safety by enforcing zero tolerance.

## Figures and Tables

**Table 1:** Peak fit parameters for five major elements on the absorbed layer in the ssDNA that hybridized the targeted LAMP amplification products and L-cysteine.

REGIONS		BE Exp *^a^*, eV	BE Literature [16,35,36,37,38,46,47,48,49,50,51,52], eV	Relative Area Intensity	Assignment
S2p	2p3/2	160.97	~161–162	91	Au–S
		163.02	~163	150	S–H
		168.32	~168–169	363	Sulfonate group
	2p1/2	162.17		45	
		164.22		75	
		169.52		182	
N1s		399.60	399.0–399.3	186	N= conjugated
		401.2	399.9–401.00	245	>N–, N–(C=O)–N, –NH_2_
Mg1s		1305.08	1305	1749	Mg–O
P2p	2p3/2	134.00	133.9–134.2	1800	${\mathrm{PO}}_{4}^{-}$ (backbone), PBS buffer
	2p1/2	134.84	134.5	900	
C1s		284.68	284.7–286.2	8846	–C–C–, –C=, –CH, –CH_3_, carbons of aliphatic contamination.
		285.90	286.0–287.0	1921	C–N, N– C=N, C–O
		287.94	287.8–289.1	564	N–(CO)–N, N–(CO)–C

*^a^*: Using Au 4f_7/2_ peak of 83.90 eV as a reference, an accuracy of ±0.5 eV.

## Data Availability

Not applicable.

## Supplementary Materials

The following are available online at https://www.mdpi.com/article/10.3390/bios11090308/s1, file S1: Estimation of mass and molecule numbers on the working surface (with the Sauerbrey’s equation [31]).
