# Supplementary material for: In-Situ Monitoring of Real-Time Loop-Mediated Isothermal Amplification with QCM: Detecting Listeria monocytogenes
Source: Biosensors (Basel). 2021 Aug 31;11(9):308. doi: 10.3390/bios11090308 (PMC8470657; doi:10.3390/bios11090308)
Supplement: Supplementary file 1 [file biosensors-11-00308-s001.zip › biosensors-1327291-supplementary.pdf]

biosensors

MDPI

Supplementary Information

In-situ Monitoring of Real-Time Loop-Mediated Isothermal Amplification with QCM: Detecting *Listeria monocytogenes*

Sirirat Wachiralurpan [1,*], Isaratat Phung-On [1], Narong Chanlek [2] Supatra Areekit [3,4], Kosum Chansiri [4,5] and Peter A. Lieberzeit [6,*]

1 Maintenance Technology Center, Institute for Scientific and Technological Research and Services, King Mongkut's University of Technology Thonburi, 126 Prachautit Rd., Bangkok 10140, Thailand; sirirat.wac@gmail.com, isaratat.phu@kmutt.ac.th
2 Synchrotron Light Research Institute (Public Organization), 111 University Avenue, Muang District, Nakhon Ratchasima 30000, Thailand; narong@slri.or.th
3 Innovative Learning Center, Srinakharinwirot University, Sukhumvit 23, Bangkok 10110, Thailand; supatraa@g.swu.ac.th
4 Center of Excellence in Biosensors, Panyananthaphikkhu Chonprathan Medical Center, Srinakharinwirot University, 222 Moo1 Tiwanon Rd., Pakkred district, Nonthaburi 11120, Thailand; supatraa@g.swu.ac.th, prof.kosum@gmail.com
5 Department of Biochemistry, Faculty of Medicine, Srinakharinwirot University, Sukhumvit 23, Bangkok 10110, Thailand; prof.kosum@gmail.com
6 Department of Physical Chemistry, Faculty for Chemistry, University of Vienna, Waehringer Strasse 42, Vienna 1090, Austria; peter.lieberzeit@univie.ac.at
* Correspondence: sirirat.wac@gmail.com (S.W.); peter.lieberzeit@univie.ac.at (P.A.L.)

1. Estimation of mass and molecule numbers on the working surface.

The irreversible signal (*ΔF*, Hz), is given in Eq. (1) according to the Sauerbrey's equation [47].

$$\Delta F = -\frac{2F0^2}{A\sqrt{\rho_q \mu_q}} \Delta m \quad (1)$$

For the mass change (*Δm*, g), is given in Eq. (2)

$$\Delta m = -\frac{\Delta F \, . \, A}{S_Q} \quad (2)$$

where ΔF is the measured irreversible frequency shift, Δm the mass change (g), A the electrode area 0.2 cm², ρ_q the density of quartz (2.648 g·cm^{-3}) and μ_q the shear modulus of quartz for AT-cut crystal (2.947x10^{11} g·cm^{-1}·s^{-2}) so that S_Q or $\rho_q \mu_q$ is 2.26x10^{8} cm^{2}g^{-1}s^{-1}, 10 MHz AT-cut Quartz was used in the study. The molecule numbers on working surface is obtained from the mass change (*Δm*) in Eq. (2) and Eq. (3), as follows

$$Number\ of\ mols = \frac{gram\ (mass)}{Molecular\ weight\ (Mw)} = \frac{number\ of\ molecules}{6.022 \times 10^{23}\ (Avogadro's\ number)}$$

$$Number\ of\ molecules = \frac{\Delta m}{Mw}\ x\ \ (6.022 \times 10^{23}) \quad (3)$$

Example; Figure 1 shows the ΔF of -342 Hz,

$$\Delta m = -\frac{\Delta f\ .\ A}{S_Q}$$

$$\therefore \Delta m = -\frac{-342 \times 0.2}{(2.26 \times 10^8)} = 0.303 \times 10^{-6}\ \text{gram} = 0.303\ \mu g$$

Therefore, the estimation of mass on working surface is 0.3 µg

$$Number\ of\ molecules = \frac{\Delta m}{Mw} \times (6.022 \times 10^{23})$$

$$\therefore molecule = \frac{0.3x\ 10^{-6}}{9982.52}\ (6.022\ x\ 10^{23}) = 1.81 \times 10^{13}\ \text{molecules}$$

Therefore, the number of molecules of ssDNA probe on surface contain approximately 1.8 × 10^{13} molecules.
